# Fear Avoidance after Concussion Tool (FACT): patient-reported outcome measure development and content validation

**DOI:** 10.1136/bmjsem-2025-002811

**Published:** 2025-11-11

**Authors:** Liam J Sherwood, Gill Cowen, Mandy Vidovich, Jeanette Hasleby, Jon Patricios, Myles C Murphy

**Affiliations:** 1 Nutrition and Health Innovation Research Institute, School of Medical and Health Sciences, Edith Cowan University, Joondalup, Western Australia, Australia; 2 School of Medicine, The University of Notre Dame Australia, Fremantle, Western Australia, Australia; 3 The Rural Clinical School of Western Australia, University of Western Australia, Broome, Western Australia, Australia; 4 Curtin Medical School, Curtin University, Bentley, Western Australia, Australia; 5 Curtin School of Population Health, Curtin University, Bentley, Western Australia, Australia; 6 Curtin Medical Research Institute, Curtin University, Bentley, Western Australia, Australia; 7 Mandy Vidovich and Associates, Subiaco, Western Australia, Australia; 8 Patient and Public Involvement Representative, Perth, Western Australia, Australia; 9 Wits Institute for Sport and Health (WISH), University of the Witwatersrand Faculty of Health Sciences, Johannesburg, South Africa; 10 Institute for Health Research, The University of Notre Dame Australia, Fremantle, Western Australia, Australia

**Keywords:** Concussion, Brain, Psychology, Measurement

## Abstract

**Objective:**

No valid patient-reported outcome measures (PROMs) exist to evaluate fear avoidance behaviour following concussion. This study developed a new PROM to evaluate fear avoidance behaviour following concussion and assessed the PROM content validity.

**Design:**

PROM development was performed using a qualitative study, and content validation was performed using a mixed-methods approach.

**Methods:**

The development phase consisted of compiling potentially relevant items (eg, questions) from other PROMs proposed to measure fear avoidance before proceeding to a series of one-on-one semistructured interviews with patient and professional participants. The content validity (relevance, comprehensibility and comprehensiveness) of the PROM was first assessed by professional participants using an online survey, before further assessment by patient participants during one-on-one semistructured interviews.

**Results:**

For PROM development, seven patient participants (n=4 men) and seven professional participants (n=4 men) from six clinical professions were included, who generated 119 potentially relevant items under seven distinct domains. Key themes that emerged during development were a strong preference for a short PROM, with a simple scale, that has clinical utility. 38 professional participants (47% women) and seven patient participants completed the content validation. Thus, the final PROM (Fear Avoidance after Concussion Tool or FACT) has content validity, consists of 28 items, with four items per domain. The seven domains of the FACT are: general, physical, psychological, hypervigilance, cognitive, social and work.

**Conclusion:**

The FACT has been developed to evaluate fear avoidance behaviour following concussion and has content validity. Future research should evaluate other measurement properties before implementation.

**WHAT IS ALREADY KNOWN ON THIS TOPIC**

- No current patient-reported outcome measures to assess fear avoidance following concussion are valid.

**WHAT THIS STUDY ADDS**

- The Fear Avoidance after Concussion Tool (FACT) has been developed with patient and professional participants.
- The FACT has content validity (relevance, comprehensiveness, comprehensibility) for evaluating fear avoidance following concussion.

**HOW THIS STUDY MIGHT AFFECT RESEARCH, PRACTICE OR POLICY**

- The FACT can be used in research and clinical practice, but caution should be taken without further evaluation of measurement properties.

## Background

 A concussion is a traumatic brain injury caused by biomechanical force transmitted to the brain, resulting in temporary neurological dysfunction that does not appear on routine neuroimaging.^1^ While many adults make a full symptomatic recovery within 7–10 days, a proportion (10–25%) will experience persisting (defined as more than 4 weeks) symptoms.^2^ People with persisting postconcussion symptoms (PPCS) present with a range of cognitive (eg, difficulty concentrating), emotional (eg, low mood), somatic (eg, headache) and sleep-associated symptoms.^3^

Why some people have persisting symptoms, and others do not, is not fully understood. However, contemporary research has recognised that there are a number of psychological factors that can increase an individual’s vulnerability to experiencing persisting symptoms. Anxiety is a psychological construct that is common in chronic health conditions,^4^ such as lower back pain or tendinopathy,^5^ and is proposed to be associated with symptom severity. One explanation for why anxiety is linked to a poorer prognosis is that anxiety may predispose people to maladaptive coping strategies, such as fear avoidance behaviour.^6^ Fear avoidance behaviour prevents desensitisation to potentially noxious stimuli.^7^ Even in the absence of symptoms, the anticipation of an exacerbation of symptoms or reinjury can lead to further fear avoidance behaviour.^7^ By abstaining from activities that have previously exacerbated symptoms (eg, cognitive load or physical activity), fear avoidance can result in further inactivity and, consequently, greater disability.^7^ Thus, fear avoidance behaviour is a recognised risk factor for persisting symptoms.^8^

It is proposed that if fear-avoidant behaviour can be recognised early, it may enable targeted management for individuals who present as being at risk of PPCS. Interventions, such as early education, and targeting psychological drivers of behaviour, have been shown to improve the prognosis of persisting symptoms.^9^,^11^ These interventions aim to empower the individual to self-manage and control their condition, reducing the negative influence that fear avoidance behaviour may have on recovery.^9^ These results have also been shown in other conditions where fear avoidance behaviour is common, such as low back pain, whereby early recognition of the condition has enabled timely intervention and improved health outcomes.^12 13^

Existing patient-reported outcome measures (PROMs) that assess fear avoidance following concussion have been evaluated within a systematic review using the Consensus-based Standards for the Selection of Health Measurement Instruments (COSMIN) guidelines.^14^ Included measures such as the Fear Avoidance Short Form Scale,^15^ and Fear Avoidance Behaviour after Traumatic Brain Injury,^16^ have insufficient content validity. Therefore, there is no evidence that current PROMs accurately measure the construct they are intended to measure (fear avoidance behaviour), which can lead to erroneous clinical and research assumptions. To ensure adequate content validity, PROMs must be developed in conjunction with a diverse sample of patient (people with lived experience of concussion) and professional participants.^17^

Persisting symptoms can have life-changing impacts through unemployment,^18^ cognitive deficits and reduced quality of life.^19^ A validated PROM could be used clinically to explore potential psychological factors of persisting symptoms in individuals with high risk for fear avoidance behaviour postconcussion. This may lead to greater utilisation of early education, reassurance and support to potentially mitigate the risk of prolonged or persisting symptoms.^9^ This study aims to develop a new PROM to identify individuals who have sustained a concussion and exhibit fear avoidance behaviour.

## Methods

### Study design

A mixed-methods study was performed to develop a new PROM using a reflective model and perform subsequent content validation of that PROM with reporting of this submission in accordance with COSMIN guidelines. This involved three consecutive stages: (1) PROM development, (2) professional participant content validation and (3) patient participant content validation. This study was designed in accordance with the COSMIN guidelines^14^ and previous content validation research.^20^

### Project team

This project was led by a multidisciplinary project team (online supplemental appendix A) of experts in concussion management, concussion research and PROM development.

### Participants

This study includes both patient participants and professional participants (researchers and healthcare clinicians).

#### Patient participants

A group of adults who had sustained a concussion and experienced concussion symptoms was established through purposive sampling from clinical and personal networks of the project team. Participants required a clinical diagnosis of concussion from a medical professional. Participants were required to have sufficient English language skills, which were deemed sufficient for inclusion if they were capable of reading the participant information letter and completing the Qualtrics survey (Qualtrics, Provo, UT). Recruitment was limited to participants in Australia.

Participants were excluded if they had sustained a moderate to severe brain injury (as evidenced by changes on routine neuroimaging) or had other pre-existing neurological conditions such as neurodegenerative conditions (eg, dementia, Parkinson’s disease) or stroke.

To ensure a range of perspectives was included, we targeted a mix of concussion recovery phases, including acute (less than 2 weeks postinjury), subacute (2–4 weeks), persisting (>4 weeks) or recovered (following a period of persisting symptoms). For participants in the acute postinjury phase, considerations were made to ensure interviews were no more exhaustive than a routine concussion assessment. Participants had the options to take regular breaks if there was an exacerbation of symptoms, postpone the interview or withdraw consent at any point.

Three groups of adults (18+ years) with acute, persisting or recovered concussion from three leading causes were targeted: (1) sport-related injury, (2) workplace injury and (3) other (eg, motor vehicle accident, fall or assault).

#### Professional participants: clinical or research experience with concussion

Professional participants were recruited internationally via email to ensure we captured diverse practice in the assessment and management, irrespective of healthcare systems. Clinical experts were identified via purposive sampling. We targeted general practitioners, neurologists, neuro-optometrists, neuropsychologists, physiotherapists and sports medicine physicians. Research experts were identified using Expertscape. These methods were used to achieve diversity in the sampled professional participants.

### Phase 1: PROM development and item generation

Items were initially generated by the steering committee, guided by a systematic review,^21^ other emerging literature within the fear avoidance behaviour PROM landscape^1516 22^,^27^ and the project team knowledge. 14 semistructured, one-to-one online interviews (using Microsoft Teams) with both professional participants (n=7) and patient participants (n=7) were performed, audio recorded and transcribed verbatim. According to the COSMIN risk of bias checklist, more than six patient participants and six professional participants are considered ‘very good’ for PROM development.^28^

A live document was used to guide the interview, which included a list of items that was shared to each participant in real time. Participants were asked to suggest items they thought were relevant and these items were added in real time. The goal of the interviews was to maximise the generation of items that related to fear avoidance after concussion.

In addition, participants were asked to comment on the format of the potential PROM. This included choosing an appropriate type of scale (eg, 5-point Likert scale), their preference for electronic or paper-based questionnaires, the total number of items to be included, duration for completion and any other general comments.

One researcher (LJS) coded all data using QSR NVivo (V.12) under the guidance of senior members of the research team. The transcripts were first read line by line to identify significant statements that captured individual perspectives of the participants. Significant statements were then grouped into themes by a single researcher (LJS) and cross-checked by a senior member of the research team. These themes were then used to inform PROM development (eg, the number of items to be included or the preferred scale to be used).

The provisional PROM generated from this stage was then reviewed for formatting and typographical errors by the research team for refinement so that a single-draft PROM was available for phase 2.

### Phase 2: professional participant content validity

Participants in phase 2 were emailed with a link to Qualtrics (Qualtrics, Provo, UT), which was used for consent and to complete the online questionnaire. The draft PROM from phase 1 was sent to 89 professional participants, identified by the project committee as recognised clinical or research experts in the field of concussion. We continued to recruit until we achieved the minimum required number to be considered adequate for content validity (>30 participants), as per COSMIN recommendations.^14^ Participants were asked to rate each item in the PROM for relevance using a 4-point scale of: strongly disagree, disagree, agree or strongly agree. Each item was voted on if it was deemed comprehensible (yes/no) before commenting on whether the PROM was overall comprehensive.

#### Relevance

Consensus for inclusion was achieved when an item reached 75% of scores being rated as agree or strongly agree, a commonly used cut-off in the literature and one that has been used in previous research.^20 29^

#### Comprehensibility

If an item was unclear, the participant was asked for ideas to improve the items’ comprehensibility. Consensus for inclusion was again achieved when an item reached 75% of scores being ‘yes’.^20 29^

#### Comprehensiveness

Participants were asked to comment on whether the overall PROM was comprehensive and whether further items were required to be included, with participant responses entered as free text.

#### Content validation

The criteria for having acceptable content validity for an item were:

- Consensus achieved for relevance.
- Consensus achieved for comprehensibility.
- There was no inclusion of additional items (beyond those already included in the PROM) when evaluated for comprehensiveness.

### Phase 3: patient participant content validity

In this phase, patient participants were asked to fill out the PROM developed in phase 2 via Qualtrics (Qualtrics, Provo, UT), and completion times were recorded. Patient participants then completed a 1:1 interview via telephone or videoconference, depending on participant preference. This interview was then guided by the PROM. Participants were specifically asked about the relevance, comprehensibility and comprehensiveness. The relevance was assessed by asking whether each item was applicable to the patient’s experience. The comprehensibility was assessed by asking the participant what they thought each item was asking, and a judgement was made by the researcher that this matched the intent of the item. Comprehensiveness was judged by asking participants whether anything was missing, and whether anything else needed to be considered for the PROM.

## Results

### Phase 1

#### Patient participants

We had seven participants with a lived experience of concussion (four men and three women) with a mean (SD) age of 36.4 (15.4) years. All seven participants recorded being Caucasian. Two participants were multilingual, with the remaining five speaking only English. Two participants’ highest level of education was graduating high school, four had obtained a bachelor’s degree and one had a professional degree (ie, medical doctor). Six of the seven participants were working full time. A previous mental health diagnosis was reported in four participants. The median number of concussions experienced by each participant was 1, with the number of concussions ranging from 1 to 9. Regarding each participant’s most recent concussion experience, four occurred during sport, two in a motor vehicle accident and one in the workplace.

#### Professional participants

We included seven professional participants (four men, three women), representing general practice, neurology, neuro-optometry, neuropsychology (two participants), physiotherapy and sports medicine. All participants were currently practising in Australia.

#### Themes

Patient and professional participants affirmed seven domains of fear avoidance: general, physical, psychological, catastrophising, cognitive, social, work. Exemplar quotations are provided in online supplemental appendix B. A prominent theme was to remove the university/school domain because participants thought this would fall under ‘cognitive’ and would not encapsulate the people not studying at a university or school at the time of injury. One participant stated:

“It doesn’t matter whether it’s work or school. The avoidance around cognitive stuff is specific to cognitive activity, regardless of the environment.” (Professional participant 4)

Thus, the university/school subtheme was removed. Participants highlighted the need for a short PROM (less than 30 items total), taking less than 5 min to complete. Participants indicated no clear preference for having the PROM as either electronic or paper (table 1).

**Table 1:** Themes related to PROM development

Theme	Exemplar quotation
Scale	“I definitely agree that you should have a four-point scale, not a five.” (Professional participant 2)
Electronic versus paper	“I think an electronic copy is better as long as we’re happy that somebody can complete it for them, and that’s not going to change the validity of the questionnaires.” (Professional participant 2)
	“It would always be my preference to have both electronic and paper copies.” (Professional participant 4)
Duration to complete	“In this kind of world, people don’t have more than 5 minutes.” (Patient participant 1)
Clinical utility	“They (concussed people) still would not have symptoms if all of this stuff was captured and picked up early on.” (Professional participant 4)
Comprehensibility	“It needs to be very easy for them to understand and do, because otherwise if it’s like too complicated, they just give up.” (Professional participant 5)

PROM, patient-reported outcome measure.

### Phase 2

We had 38 professional participants (42% response rate) with a mean age of 45 (range 27–75) years responding to our survey (table 2). Participants also reported their ethnicity, which assisted us in our evaluation of sample diversity. However, because this was a free text option and used only to ensure a diverse sample, these data were not reported, as responses and classifications were extremely varied. Geographically, there were five continents, and nine countries represented. Of the respondents, 21 (55%) held a doctoral degree, 8 (21%) held a professional degree (Medical Doctor or Juris Doctor), 5 (13%) held a master’s degree and 4 (11%) held a bachelor’s degree. Researchers were recruited if their expertise was either in concussion, fear avoidance or PROM development, and clinicians were recruited with expertise in concussion.

**Table 2:** Professional participant demographic data

Domain	Variables	n (%)
Gender	Woman	18 (47)
	Man	19 (50)
	Prefer not to say	1 (3)
Country of origin	Australia	25 (65)
	Canada	3 (8)
	United States of America	2 (5)
	South Africa	2 (5)
	New Zealand	2 (5)
	Switzerland	1 (3)
	Italy	1 (3)
	United Kingdom	1 (3)
	Qatar	1 (3)
Highest level of education	Bachelor’s degree	4 (11)
	Master’s degree	5 (13)
	Doctoral degree	21 (55)
	Professional degree (MD or JD)	8 (21)
Employment status	Working full time	33 (87)
	Working part-time	2 (5)
	Student	2 (5)
	Other	1 (3)

JD, Juris Doctor; MD, Medical Doctor.

#### Relevance

A total of 119 items were sent to experts via Qualtrics (Qualtrics, Provo, UT, online supplemental appendix C). Of these items, 28 did not reach the 75% a priori cut-off and were deemed not relevant. The top items in each domain are listed in online supplemental appendix D.

#### Comprehensibility

All items that were deemed relevant met the criteria for comprehensibility (online supplemental appendix D). However, some minor amendments were based on suggested language from professional participants.

We opted to omit certain items based on similarity to higher scoring items (eg, the content of the item was the same but worded in different ways—in which case we deferred to the items with a higher comprehensibility rating). As mentioned in phase 1, we removed university/school as a domain given the thematic analysis in phase 1 as well as the similarity of items with the cognitive domain. We also opted to include ‘I avoid interacting on social medial because it will make my symptoms worse’ over ‘I avoid going to the shops because they will make my symptoms worse’ to ensure we encompass socialising physically as well as via internet.

### Phase 3

The final PROM was first sent to members of the research team for checking before being sent to patient participants. Of the items that met the criteria for content validity, based on the patient participants’ stated preference for PROM length in phase 1, only the top four per domain for relevance (as judged by the combined number of agree and strongly agree scores) were included in the PROM. After research team evaluation, we sent the PROM to the original seven patient participants from phase 1. However, while they all completed the PROM, three were unavailable to participate in the one-on-one interview. Thus, a further three participants were sampled and completed both the PROM and the interview. Thus, for phase 3, 10 patient participants were included, with ages ranging from 22 years to 68 years, and the total number of concussions ranging from 1 to 9. The newly included participants represented the same group of phase 1 participants who were unavailable to participate (two recovered from persisting symptoms postconcussion, one subacute).

All seven participants who completed both the PROM and the interview reported that the final included items in the Fear Avoidance after Concussion Tool (FACT) PROM were relevant and comprehensible. Further, patient participants reported that the length of the PROM was satisfactory and comprehensive. A concern from multiple patient participants was the use of the word ‘catastrophising’ to describe one of the domains and the impact this may have on the person completing the questionnaire. Thus, patient participants suggested the domain name be changed to ‘hypervigilance’, and this recommendation was accepted. The median (IQR) time to complete the questionnaire was 3 min and 54 s (4 min and 13 s), with the minimum completion time being 2 min and 28 s, and the maximum completion time being 28 min and 21 s. The final FACT is presented in table 3.

**Table 3:** Fear Avoidance after Concussion Tool (FACT)

Please answer the following questionnaire according to experiences you have noticed since your concussion. This should be your own views and not the view of others. Each statement should be scored from strongly disagree to strongly agree by checking the appropriate box.

Items	Strongly disagree	Disagree	Agree	Strongly agree
	0	1	2	3
General				
I avoid my usual activities.				
I put parts of my life on hold.				
I avoid certain environments (bright, loud, fast moving).				
I avoid screen time.				
Physical				
I make my symptoms worse if I return to my previous activities of daily living.				
I make my symptoms worse if I increase my physical activity.				
I make my symptoms worse if I exercise.				
I make my symptoms worse if I move too quickly.				
Psychological				
I avoid talking about my symptoms.				
I avoid talking about my recovery postconcussion.				
I avoid environments that I associate with symptom exacerbation (eg, shopping centres, stadiums).				
I know my injury has impacted my mental well-being, but I avoid seeking professional care.				
Hypervigilance				
It’s not safe for a person with a concussion like mine to be physically active.				
I do not do all the things I used to do because it’s too risky since my concussion.				
I worry what my life will be like if my symptoms do not improve.				
I will always have symptoms of concussion.				
Cognitive				
I spend more time resting than doing cognitive activities (eg, tasks that involve increased concentration) because of my symptoms.				
I avoid doing mental activities as it makes my symptoms worse.				
When I have my symptoms, thinking or concentrating will make the symptoms worse.				
I stop the mental activity I am doing when my symptoms are worsening.				
Social				
I avoid social settings that will make my symptoms worse.				
I avoid catching up with friends and family because it will make my symptoms worse.				
I avoid leaving my house because it will make my symptoms worse.				
I avoid social media because it will make my symptoms worse.				
Work				
I avoid physical jobs because it will increase my symptoms.				
I fear that my symptoms will result in poor workplace performance.				
I fear that my symptoms will cause me to lose my job.				
I should not do my normal work with my symptoms.				

A total score is calculated for the domain (out of 12) and the entire patient-reported outcome measure (PROM) (out of 84).

## Discussion

This study aimed to develop a PROM to be completed by people who sustained a concussion to measure fear avoidance behaviour. A reflective model was used to develop the PROM that measures the construct of fear avoidance in the context of concussion. Therefore, items within each domain contribute to the overall construct of fear avoidance, but are distinct from the other domains. The methodology reflects the COSMIN guidelines, and methods reported in recently developed PROMs, to ensure the creation of the PROM is patient informed, with relevance, comprehensiveness and comprehensibility. The PROM we developed, termed FACT, has seven domains that each consist of four items. We also aimed to create a PROM that uses language that is easy to understand, and that the PROM is quick to administer in a clinical setting, given the themes identified within phase 1 and phase 3 with patient participants.

Previous PROMs that measure fear avoidance in the field of concussion have been adapted from musculoskeletal conditions and have been shown to have inadequate validity due to the lack of patient and professional participant involvement in their development. International guidelines still recommend the use of PROMs in other fields of sports medicine,^30^ despite lacking content and construct validity.^31 32^ Continuous use of PROMs with poor measurement properties in research will lead to erroneous conclusions. Our methodology ensured that the FACT was developed with adequate content validity, so it is specifically relevant to the concussion population and their potential fear avoidance behaviour.

We developed the FACT in three phases: phase 1—to generate relevant, comprehensible items and achieve comprehensiveness; phase 2—to evaluate relevance, comprehensibility and comprehensiveness using international professional participants; and phase 3—to ensure relevance, comprehensibility and comprehensiveness from a patient perspective. The majority of items generated in phase 1 were deemed relevant by the professional group. The patient preference was for a PROM of 20–30 items and to take no longer than 5 min to administer. We opted to uphold this recommendation to minimise the risk of symptom exacerbation that may occur due to prolonged periods of concentration.

We omitted the university/school domain and then chose the most relevant four items from all other domains to ensure the PROM was developed in accordance with patient and professional suggestions. We wanted to develop a PROM relevant for the general population, given that those studying between the ages of 15 and 74 only make up 16% of the total Australian population.^33^ Additionally, the items within the university/school domain were adequately covered in other domains. We opted to remove specific items (eg, removing the items related to driving) for the same reason, given they were not always relevant to the broader population. Measurement invariance is a psychometric property of PROMs that ensures the construct (fear avoidance behaviour) is measured similarly across different groups or individuals. Thus, personal factors other than the concussion, such as currently studying or ability to drive, can result in systemic differences in how people answer.

The final items were all deemed comprehensible using the 75% cut-off. However, the research team opted to amend the items as per professional participant suggestions (even when comprehensibility was achieved). Thus, other than amending the catastrophising domain, no further wording changes were required when reviewed by patient participants in stage 3.

We reached saturation of items (119) in phase 1 from 14 interviews. This is a substantial number of items, likely to increase respondent burden. This longer completion time can lead to disengagement and reduce the accuracy of responses. Further, this included substantially more items than a near-identical process performed in Achilles tendinopathy to develop a new outcome measure evaluating tendon-related disability (three domains, with <20 items).^20^ This suggests that the breadth of concussion symptoms and potentially aggravating activities identified in phase 1 is much broader than a common musculoskeletal presentation, which may account for the large number of items initially generated.

Fear avoidance behaviour may be associated with adverse clinical outcomes following concussion, such as persisting concussion symptoms or functional neurological disorder.^15 34^ Both persisting concussion symptoms and functional neurological disorder have similar symptom profiles, with the presence of cognitive and somatic symptoms.^34^ Subsequently, functional neurological disorder may be under-recognised following head injury.^34^ It is likely people with either persisting concussion symptoms or functional neurological disorder exhibit fear avoidance behaviours. However, prior studies evaluating the prevalence of fear avoidance in people following concussion have used invalid outcome measures, and the accuracy of these results is likely to be questionable. We propose that using the FACT to identify individuals who exhibit fear avoidance is important as they could serve as potential targets for early education and/or psychological intervention to reduce such behaviours. This may lead to increased concussion education, which is important in alleviating stress associated with the diagnosis and prognosis.^35^ This could be tailored to each individual based on clinical judgement by addressing the items that the patient scores highly in. For example, if the patient scores highly in the physical section, more emphasis could be placed on creating a management plan that involves progressive increases in exercise.^36^ It is still unknown as to what point this PROM is best administered in recovery. However, we propose that it would be most beneficial if administered early in an individual’s recovery before symptoms are classified as ‘persisting’ (28 days).

The biggest strength of this study is in the methodological rigour. Our methodology allows the balance of both patient and professional input into the development of the PROM, ensuring adequate content validity. The sample sizes used were in keeping with ‘very good’ for PROM development and ‘very good’ or ‘adequate’ for content validation. Thus, we created a PROM that is designed to measure the construct of fear avoidance in individuals who have sustained a concussion.

This research represents a development and content validation study and has not evaluated construct validity, internal consistency and reliability, which are essential to widespread adoption of the PROM. However, no other PROM has content validity for evaluating fear avoidance following concussion. COSMIN recommendations state that all other measurement properties cannot exceed the judgement of content validity. Therefore, despite not having all measurement properties confirmed, the FACT currently represents the PROM with the highest level of validity and may be used. This is due to other PROMs measuring the construct of fear avoidance following concussion judged as poor with insufficient development and a lack of content validation.^21^

## Conclusion

We developed the FACT, a PROM to be used in research and clinical settings, to assist in the management of patients with concussion by identifying those patients with maladaptive fear avoidance. The FACT contains seven subscales (general, physical, psychological, hypervigilance, cognitive, social, work) comprising 28 items, which take approximately 3.5 min to complete. Content validity was confirmed by both patient and professional participants. Future research should establish other essential measurement properties of the FACT, such as reliability and construct validity.

## Supplementary material

10.1136/bmjsem-2025-002811 online supplemental file 1

## Data Availability

No data are available.

## References

[1] Patricios JS, Schneider KJ, Dvorak J (2022). Consensus statement on concussion in sport: the 6th International Conference on Concussion in Sport-Amsterdam, October 2022. Br J Sports Med.

[2] Scott OFT, Bubna M, Boyko E (2023). Characterizing the profiles of patients with acute concussion versus prolonged post-concussion symptoms in Ontario. Sci Rep.

[3] Levin HS, Diaz-Arrastia RR (2015). Diagnosis, prognosis, and clinical management of mild traumatic brain injury. Lancet Neurol.

[4] Sareen J, Cox BJ, Clara I (2005). The relationship between anxiety disorders and physical disorders in the U.S. National Comorbidity Survey. *Depress Anxiety*.

[5] White G, Bright F, Rio EK (2025). Do Anxiety, Depression, Fear of Movement and Fear of Achilles Rupture Correlate with Achilles Tendinopathy Pain, Symptoms or Physical Function?. *J Clin Med*.

[6] Santarnecchi E, Sprugnoli G, Tatti E (2018). Brain functional connectivity correlates of coping styles. *Cogn Affect Behav Neurosci*.

[7] Gatchel RJ, Neblett R, Kishino N (2016). Fear-Avoidance Beliefs and Chronic Pain. *J Orthop Sports Phys Ther*.

[8] Wijenberg MLM, Stapert SZ, Verbunt JA (2017). Does the fear avoidance model explain persistent symptoms after traumatic brain injury?. Brain Inj.

[9] Broshek DK, De Marco AP, Freeman JR (2015). A review of post-concussion syndrome and psychological factors associated with concussion. Brain Inj.

[10] Bell KR, Hoffman JM, Temkin NR (2008). The effect of telephone counselling on reducing post-traumatic symptoms after mild traumatic brain injury: a randomised trial. *J Neurol Neurosurg Psychiatry*.

[11] Leddy JJ, Sandhu H, Sodhi V (2012). Rehabilitation of Concussion and Post-concussion Syndrome. *Sports Health*.

[12] Hetrick SE, Parker AG, Hickie IB (2008). Early identification and intervention in depressive disorders: towards a clinical staging model. Psychother Psychosom.

[13] Wand BM, Bird C, McAuley JH (2004). Early intervention for the management of acute low back pain: a single-blind randomized controlled trial of biopsychosocial education, manual therapy, and exercise. Spine (Phila Pa 1976).

[14] Prinsen CAC, Mokkink LB, Bouter LM (2018). COSMIN guideline for systematic reviews of patient-reported outcome measures. *Qual Life Res*.

[15] Silverberg ND, Panenka WJ, Iverson GL (2018). Fear Avoidance and Clinical Outcomes from Mild Traumatic Brain Injury. J Neurotrauma.

[16] Snell DL, Siegert RJ, Debert C (2020). Evaluation of the Fear Avoidance Behavior after Traumatic Brain Injury Questionnaire. *J Neurotrauma*.

[17] Murphy MC, Dijkstra HP, Ardern CL (2025). Considerations for improving patient and professional participant diversity in sports medicine, rehabilitation and sports science research. Br J Sports Med.

[18] Boake C, McCauley SR, Pedroza C (2005). Lost productive work time after mild to moderate traumatic brain injury with and without hospitalization. Neurosurgery.

[19] King NS, Kirwilliam S (2011). Permanent post-concussion symptoms after mild head injury. Brain Inj.

[20] Murphy MC, Newsham-West R, Cook J (2024). TENDINopathy Severity Assessment - Achilles (TENDINS-A): Development and Content Validity Assessment of a New Patient-Reported Outcome Measure for Achilles Tendinopathy. *J Orthop Sports Phys Ther*.

[21] Sherwood LJ, Korakakis V, Mosler AB (2023). Quantifying Fear Avoidance Behaviors in People With Concussion: A COSMIN-Informed Systematic Review. *J Orthop Sports Phys Ther*.

[22] Cairncross M, Brooks BL, Virani S (2021). Fear avoidance behavior in youth with poor recovery from concussion: measurement properties and correlates of a new scale. *Child Neuropsychol*.

[23] Cairncross M, Debert CT, Hunt C (2021). Normative Data for the Fear Avoidance Behavior After Traumatic Brain Injury Questionnaire in a Clinical Sample of Adults With Mild TBI. *J Head Trauma Rehabil*.

[24] Waddell G, Newton M, Henderson I (1993). A Fear-Avoidance Beliefs Questionnaire (FABQ) and the role of fear-avoidance beliefs in chronic low back pain and disability. *Pain*.

[25] French DJ, France CR, Vigneau F (2007). Fear of movement/(re)injury in chronic pain: a psychometric assessment of the original English version of the Tampa scale for kinesiophobia (TSK). *Pain*.

[26] Seng EK, Klepper JE (2017). Development of the Cogniphobia Scale for Headache Disorders (CS-HD): A pilot study. *Psychol Assess*.

[27] Horowitz M, Wilner N, Alvarez W (1979). Impact of Event Scale: a measure of subjective stress. *Psychosom Med*.

[28] Mokkink LB, de Vet HCW, Prinsen CAC (2018). COSMIN Risk of Bias checklist for systematic reviews of Patient-Reported Outcome Measures. *Qual Life Res*.

[29] Diamond IR, Grant RC, Feldman BM (2014). Defining consensus: a systematic review recommends methodologic criteria for reporting of Delphi studies. J Clin Epidemiol.

[30] de Vos R-J, Gravare Silbernagel K, Malliaras P (2024). ICON 2023: International Scientific Tendinopathy Symposium Consensus – the core outcome set for Achilles tendinopathy (COS-AT) using a systematic review and a Delphi study of professional participants and patients. *Br J Sports Med*.

[31] Korakakis V, Kotsifaki A, Stefanakis M (2021). Evaluating lower limb tendinopathy with Victorian Institute of Sport Assessment (VISA) questionnaires: a systematic review shows very-low-quality evidence for their content and structural validity-part I. *Knee Surg Sports Traumatol Arthrosc*.

[32] Travers N, Murphy MC, Wand BM (2025). The Victorian Institute of Sport Assessment - Achilles is fundamentally flawed and unfit for clinical practice or research: A Rasch Measurement Theory Analysis using COSMIN recommendations. Phys Ther Sport.

[33] (2024). Education and work, Australia (ABS).

[34] Burke MJ, Silverberg ND (2025). New framework for the continuum of concussion and functional neurological disorder. Br J Sports Med.

[35] Ponsford J, Willmott C, Rothwell A (2002). Impact of early intervention on outcome following mild head injury in adults. J Neurol Neurosurg Psychiatry.

[36] Katz M, Lenoski S, Ali H (2020). Concussion Office Based Rehabilitation Assessment: A Novel Clinical Tool for Concussion Assessment and Management. Brain Sci.

