# Supplementary material for: Fear Avoidance after Concussion Tool (FACT): patient-reported outcome measure development and content validation
Source: BMJ Open Sport Exerc Med. 2025 Nov 11;11(4):e002811. doi: 10.1136/bmjsem-2025-002811 (PMC12606469; doi:10.1136/bmjsem-2025-002811)
Supplement: online supplemental file 1 [file bmjsem-11-4-s001.docx]

**SUPPLEMENTARY MATERIAL**

**Appendix A. Project Committee**

| **Appointment** | **Name** | **Role** | **Qualifications** |
| --- | --- | --- | --- |
| Steering Committee | Liam Sherwood | Masters by research student; clinical physiotherapist | Bachelor of Physiotherapy |
| Steering Committee | Myles Murphy | Physiotherapy clinician researcher | Bachelor of Physiotherapy, Graduate Certificate in Sports Physiotherapy, Master of Clinical Physiotherapy (Sports), PhD (Physiotherapy), greater than 10 years’ experience in sports physiotherapy. |
| Steering Committee | Lauren Fortington | Injury epidemiologist | PhD and research experience in concussion research and epidemiology. |
| Project Committee | Gill Cowen | General practitioner clinician researcher. | Bachelor of Medicine, Bachelor of Surgery (MBBS), Master of Arts, Fellowship of RACGP, Master of Sports Medicine, Graduate Certificate in Health Leadership, greater than 20 years’ experience as a General Practitioner and Sports Doctor. |
| Project Committee | Mandy Vidovich | Neuropsychologist clinician researcher. | Bachelor of Science (Psychology) with Honours, Masters in Neuropsychology, PhD (Neuropsychology), greater than 25 years of clinical experience in Neuropsychology. |

**Appendix B. Sub-domain themes.**

| **Themes** | **Quote** |
| --- | --- |
| **General** | **“**You're not having to worry about work, school, university, because the things that in that environment that often are triggering for people that I see relate to having to concentrate noise stimuli in the environment.” (Professional Participant 4) |
| **Physical** | **“**For me it's been sport. I've avoided footy. I haven't gone back to that and I'm not sure if I will.” (Patient Participant 5) |
| **Psychological** | **“**you're also going to get a group of people where shame is a big, you know, a quite a salient factor for them.” (Professional Participant 4) |
|  | “When my girlfriend asks, ‘are you okay or how's your head?’ I'll just say ‘yeah, fine.’ I guess I avoid telling her that I actually do have pretty bad headache or feeling anxious.” (Patient Participant 5) |
|  | **“**The fear of judgment and misunderstanding of my injury.” (Patient Participant 6) |
| **Catastrophising** | **“**I'm concerned about the terminology, even though it might well be what is used (catastrophizing) from a consumer point of view.” (Professional Participant 2) |
|  | **“**People might get offended if they saw this and said that we're putting CTE in the catastrophizing category.” (Professional Participant 3) |
| **Cognitive** | “Daily learning because I haven't been doing that. I thought that might be too stressful on my brain.” (Patient Participant 5) |
| **Social** | “The concussion symptoms; how they affect my relationships.” (Patient Participant 5) |
| **Work** | “Focus more on just the cognitive and physical side of things rather than having stuff that's specific to work.” (Professional Participant 4) |
| **University/School** | “I assume that work and school would fold in together.” (Professional Participant 4)  “The avoidance around cognitive stuff is specific to doing cognitive activity, regardless of the environment.” (Professional Participant 4)  “Work and school would sit under the physical and cognitive domain potentially.” (Professional Participant 2) |

**Appendix C. Complete Item List - Relevance**

| Items | Not Answered | Strongly Disagree | Disagree | Agree | Strongly Agree |
| --- | --- | --- | --- | --- | --- |
| **General Items** | | | | | |
| I avoid my usual activities. | 15% | 0% | 0% | 30% | 55% |
| I put parts of my life on hold. | 15% | 0% | 6% | 36% | 42% |
| I avoid certain environments (bright, loud, fast-moving) because they worsen my symptoms. | 15% | 0% | 9% | 33% | 42% |
| I avoid driving due to fear of re-injury. | 15% | 0% | 12% | 30% | 42% |
| I avoid screentime to minimise the risk of exacerbating my symptoms. | 15% | 0% | 12% | 33% | 39% |
| I avoid loud music because it will increase my concussion symptoms. | 15% | 0% | 15% | 36% | 33% |
| I avoid drinking alcohol because it will make my symptoms worse. | 15% | 3% | 12% | 42% | 27% |
| I need to get imaging of my brain because something is dangerously wrong. | 15% | 0% | 18% | 18% | 48% |
| I am fearful of not having good sleep quality. | 15% | 0% | 21% | 42% | 21% |
| I avoid foreign languages and subtitles because they make my symptoms worse. | 15% | 3% | 52% | 21% | 9% |
| **Physical** | | | | | |
| I'm afraid that I might make my symptoms worse if I exercise. | 12% | 0% | 3% | 18% | 67% |
| I'm afraid that I might make my symptoms worse if I return to my previous activities of daily living. | 12% | 0% | 3% | 39% | 46% |
| I'm afraid that I might make my symptoms worse if I increase my physical activity. | 12% | 0% | 6% | 30% | 52% |
| I avoid moving too quickly because it worsens my concussion symptoms. | 12% | 0% | 9% | 36% | 42% |
| I avoid certain environments that are loud because it worsens my concussion symptoms. | 12% | 3% | 6% | 36% | 42% |
| I avoid high intensity exercise because it will make my symptoms worse. | 12% | 0% | 12% | 27% | 48% |
| I avoid environments that are bright because it worsens my concussion symptoms. | 12% | 3% | 9% | 33% | 42% |
| I avoid activities that make me feel nauseous (such as driving). | 12% | 0% | 12% | 36% | 42% |
| I avoid exercises with head movements because it will increase my symptoms. | 15% | 0% | 12% | 24% | 48% |
| I avoid contact sport due to fear of future concussion. | 12% | 0% | 15% | 36% | 36% |
| My concussion symptoms let me know when to stop exercising so that I don’t worsen my symptoms. | 12% | 0% | 15% | 39% | 33% |
| My concussion symptoms let me know what my activity level limit is so that I don’t worsen my symptoms. | 12% | 0% | 15% | 45% | 27% |
| I avoid running because it will make my symptoms worse. | 12% | 0% | 15% | 39% | 33% |
| I avoid contact sport due to fear of re-injury. | 12% | 0% | 21% | 27% | 39% |
| I need to plan my activities otherwise I will exacerbate my symptoms. | 12% | 0% | 21% | 30% | 36% |
| I avoid spontaneous activities because it will exacerbate my symptoms. | 12% | 0% | 21% | 33% | 33% |
| My concussion symptoms let me know when to stop exercising so that I don’t injure myself. | 12% | 0% | 24% | 42% | 21% |
| I avoid ball sports due to fear of re-injury. | 12% | 0% | 27% | 21% | 39% |
| **Psychological** | | | | | |
| I avoid external reminders that have previously exacerbated my concussion symptoms (e.g., shopping centres, stadiums.) | 3% | 0% | 9% | 42% | 45% |
| I avoid talking about my recovery post-concussion. | 0% | 3% | 9% | 52% | 36% |
| I avoid talking about my concussion symptoms. | 3% | 0% | 12% | 36% | 48% |
| I know my injury had impacted my mental wellbeing, but I avoid seeking professional care. | 6% | 0% | 12% | 42% | 39% |
| I avoid talking about my concussion. | 3% | 3% | 12% | 48% | 33% |
| I avoid replaying the injury in my head | 3% | 0% | 15% | 64% | 12% |
| I avoid talking about my concussion because people don't understand how I am feeling. | 3% | 3% | 12% | 55% | 27% |
| I am aware of my feelings about the concussion, but I do not deal with them. | 3% | 0% | 15% | 55% | 27% |
| I avoid the thought of my concussion. | 3% | 0% | 18% | 42% | 36% |
| I avoid talking about how severe my concussion symptoms are. | 3% | 0% | 18% | 42% | 36% |
| I avoid external reminders of a stressful experience (e.g., people, places, objects, conversations, activities, situations.) | 0% | 0% | 21% | 51% | 27% |
| I avoid seeking professional help because I am not getting better anyway. | 3% | 0% | 21% | 30% | 45% |
| I avoid seeing my doctor because they don't believe me anyway. | 3% | 0% | 33% | 27% | 36% |
| I avoid environments that I could witness a concussion. | 0% | 3% | 33% | 45% | 18% |
| I avoid talking about my concussion because people don't believe me anyway. | 3% | 0% | 36% | 48% | 12% |
| **Catastrophising Items** | | | | | |
| It's really not safe for a person with a condition like mine to be physically active. | 3% | 0% | 6% | 33% | 58% |
| I can't do all the things I used to be able to do because I worry I am more at risk of getting injured. | 3% | 0% | 6% | 42% | 48% |
| I worry how I would live if my symptoms don't improve. | 3% | 3% | 6% | 42% | 45% |
| I am worried that I will always have symptoms of concussion. | 3% | 3% | 9% | 30% | 55% |
| I can't do all the things I used to be able to do because I worry I will increase my symptoms. | 3% | 3% | 9% | 36% | 48% |
| I am fearful of the quality of life I will have if my symptoms don't improve. | 3% | 3% | 9% | 36% | 48% |
| I wouldn't have this much pain if there weren't something potentially dangerous going on in my head. | 3% | 0% | 12% | 42% | 42% |
| I am worried that I will always be like this. | 3% | 6% | 9% | 18% | 64% |
| My concussion symptoms are telling me that I have something dangerously wrong. | 3% | 0% | 18% | 15% | 64% |
| I am at risk of brain damage or worse due to my injury. | 3% | 3% | 15% | 21% | 58% |
| I am at risk of dementia due to my injury. | 3% | 0% | 18% | 24% | 55% |
| I need to retire from contact or collision activities. | 3% | 0% | 21% | 21% | 55% |
| My headaches put my head and brain at risk for the rest of my life. | 3% | 0% | 21% | 39% | 36% |
| If I were to try to overcome it, my symptoms would get worse | 3% | 0% | 21% | 39% | 36% |
| I am fearful of future psychological distress due to my condition. | 3% | 0% | 21% | 42% | 33% |
| I am at risk of chronic traumatic encephalopathy (CTE) due to my injury. | 9% | 0% | 18% | 21% | 51% |
| If the symptoms from my injury do not resolve, I could not live like this. | 3% | 6% | 18% | 33% | 39% |
| I've only had one concussion, but I need to stop all contact/collision activities. | 3% | 0% | 30% | 21% | 45% |
| I am fearful of future suicide due to my concussion. | 3% | 0% | 30% | 33% | 33% |
| **Cognitive Items** | | | | | |
| When I have my concussion symptoms, I’m afraid that thinking/concentrating too hard will make the symptoms worse. | 12% | 0% | 9% | 51% | 27% |
| I purposely avoid doing activities that might elicit a headache. | 12% | 0% | 12% | 36% | 39% |
| I’m afraid that I might make my concussion symptoms worse by concentrating too much or being too mentally active. | 12% | 0% | 15% | 27% | 45% |
| I avoid thinking or concentrating too hard because it will elicit or increase the symptoms of my concussion. | 15% | 0% | 12% | 36% | 36% |
| I stop the activity I am doing when I sense my concussion symptoms are worsening. | 12% | 0% | 15% | 39% | 33% |
| Because of my symptoms most days I spend more time resting than doing cognitive activities. | 18% | 0% | 9% | 42% | 30% |
| I'm afraid that I might make my headache pain worse by concentrating too much or being too mentally active. | 12% | 0% | 15% | 45% | 27% |
| Performing a difficult mental task frequently brings on my concussion symptoms. | 12% | 0% | 18% | 39% | 30% |
| I avoid thinking/concentrating too hard because it causes my concussion symptoms. | 12% | 0% | 18% | 39% | 30% |
| It’s really not safe for a person with an injury like mine to engage in too much thinking and concentrating. | 12% | 0% | 21% | 30% | 36% |
| I will stop concentrating as soon as I sense worsening of my concussion symptoms. | 12% | 0% | 21% | 48% | 18% |
| I avoid doing activities that might cause cognitive fatigue. | 12% | 0% | 21% | 51% | 15% |
| I avoid cognitive activities because it will worsen my concussion symptoms. | 12% | 0% | 24% | 30% | 33% |
| I avoid doing activities that might elicit my concussion symptoms. | 12% | 3% | 21% | 36% | 27% |
| I avoid driving long distances because I am fearful of having an accident. | 12% | 0% | 27% | 33% | 27% |
| I prefer to avoid doing activities that might elicit a headache. | 12% | 0% | 27% | 48% | 12% |
| Simply being careful not to concentrate too hard or too long is the safest thing I can do to prevent my concussion symptoms from worsening. | 12% | 0% | 30% | 27% | 30% |
| I avoid getting too emotional because it will exacerbate my symptoms. | 12% | 0% | 33% | 39% | 15% |
| I avoid driving long distances because I am fearful of having an accident. | 12% | 0% | 27% | 33% | 27% |
| It’s really not safe for a person with a condition like mine to be cognitively active. | 12% | 0% | 42% | 21% | 24% |
| I avoid learning a new language because it will be too much for my brain. | 12% | 9% | 45% | 24% | 9% |
| **Social Items** | | | | | |
| I avoid social settings that will make my symptoms worse. | 12% | 0% | 6% | 27% | 55% |
| I avoid catching up with friends and family because it will make my symptoms worse. | 12% | 0% | 6% | 45% | 36% |
| I avoid leaving my house because it will make my symptoms worse. | 12% | 0% | 9% | 30% | 48% |
| I avoid busy environments because it will make my symptoms worse. | 15% | 0% | 9% | 27% | 48% |
| I avoid going to the shops because they will make my symptoms worse. | 12% | 0% | 12% | 33% | 42% |
| I avoid interacting on social media because it will make my symptoms worse. | 12% | 0% | 15% | 36% | 36% |
| I avoid leaving my bedroom because it will make my symptoms worse. | 12% | 0% | 21% | 33% | 33% |
| I avoid parties because they will make my symptoms worse. | 12% | 0% | 21% | 39% | 27% |
| I avoid talking on the phone because it will make my symptoms worse. | 12% | 0% | 21% | 39% | 27% |
| I avoid environments with alcohol because it will make my symptoms worse. | 12% | 0% | 24% | 33% | 30% |
| I avoid email communication because it will make my symptoms worse. | 15% | 0% | 21% | 42% | 21% |
| I avoid going out for dinner because it will make my symptoms worse. | 15% | 0% | 24% | 36% | 24% |
| I avoid going out for coffee because it will make my symptoms worse. | 18% | 0% | 30% | 30% | 21% |
| **Work Items** | | | | | |
| I avoid physical jobs because it will increase my concussion symptoms. | 12% | 3% | 0% | 45% | 39% |
| I fear that my concussion symptoms will result in poor workplace performance. | 12% | 0% | 3% | 51% | 33% |
| I fear that my concussion symptoms will cause me to lose my job. | 12% | 0% | 6% | 39% | 42% |
| I should not do my normal work with my present symptoms. | 12% | 0% | 9% | 33% | 45% |
| I avoid work due to the stress of some tasks at work exacerbating my symptoms | 12% | 0% | 9% | 33% | 45% |
| My injury has affected my options for work. | 12% | 3% | 6% | 33% | 45% |
| I can't return to work due to lack of options for reasonable adjustments in the workplace for my injury. | 12% | 3% | 6% | 42% | 36% |
| I can't progress my workload otherwise I will exacerbate my concussion symptoms. | 12% | 0% | 9% | 45% | 33% |
| I should not ever return to work due to the risk of re-injury. | 12% | 3% | 12% | 55% | 18% |
| I should not ever return to work due to the risk of exacerbating my symptoms. | 12% | 3% | 18% | 27% | 39% |
| I can't return to work due to lack of workplace understanding of my injury. | 12% | 0% | 21% | 30% | 36% |
| I avoid my workplace because of the lack of workplace awareness of my injury. | 12% | 0% | 21% | 42% | 24% |
| My work might harm my brain. | 12% | 0% | 24% | 42% | 21% |
| I must change jobs from outdoor employment because I cannot tolerate it from a concussion point of view. | 12% | 0% | 33% | 42% | 12% |

**Appendix D. Relevance of items as judged by professional participants (n=32).**

| **Included Items** | **Relevance** | | | | **Comprehensibility** | |
| --- | --- | --- | --- | --- | --- | --- |
|  | **Strongly Disagree** | **Disagree** | **Agree** | **Strongly Agree** | **Yes** | **No** |
| **General** | | | | | | |
| I avoid my usual activities. | 0% | 0% | 36% | 64% | 93% | 7% |
| I put parts of my life on hold. | 0% | 7% | 43% | 50% | 93% | 7% |
| I avoid certain environments (bright, loud, fast-moving) because they worsen my symptoms. | 0% | 11% | 39% | 50% | 100% | 0% |
| I avoid driving due to fear of re-injury.^a^ | 0% | 14% | 36% | 50% | 95% | 7% |
| I avoid screentime to minimise the risk of exacerbating my symptoms. | 0% | 15% | 39% | 46% | 100% | 0% |
| **Physical** | | | | | | |
| I'm afraid that I might make my symptoms worse if I exercise. | 0% | 3% | 21% | 76% | 100% | 0% |
| I'm afraid that I might make my symptoms worse if I return to my previous activities of daily living. | 0% | 3% | 45% | 52% | 89% | 11% |
| I'm afraid that I might make my symptoms worse if I increase my physical activity. | 0% | 7% | 34% | 59% | 93% | 7% |
| I avoid moving too quickly because it worsens my concussion symptoms. | 0% | 11% | 41% | 48% | 100% | 0% |
| **Psychological** | | | | | | |
| I avoid external reminders that have previously exacerbated my concussion symptoms (e.g., shopping centres, stadiums.) | 0% | 9% | 44% | 47% | 75% | 25% |
| I avoid talking about my recovery post-concussion. | 3% | 9% | 52% | 36% | 88% | 12% |
| I avoid talking about my concussion symptoms. | 0% | 12% | 38% | 50% | 100% | 0% |
| I know my injury had impacted my mental wellbeing, but I avoid seeking professional care. | 0% | 13% | 45% | 42% | 81% | 19% |
| **Catastrophising** | | | | | | |
| It's really not safe for a person with a condition like mine to be physically active. | 0% | 7% | 34% | 59% | 97% | 3% |
| I can't do all the things I used to be able to do because I worry I am more at risk of getting injured. | 0% | 6% | 44% | 50% | 93% | 7% |
| I worry how I would live if my symptoms don't improve. | 3% | 6% | 44% | 47% | 97% | 3% |
| I am worried that I will always have symptoms of concussion. | 4% | 9% | 31% | 56% | 100% | 0% |
| **Cognitive** | | | | | | |
| When I have my concussion symptoms, I’m afraid that thinking/concentrating too hard will make the symptoms worse. | 0% | 10% | 59% | 31% | 96% | 4% |
| I purposely avoid doing activities that might elicit a headache. | 0% | 14% | 41% | 45% | 86% | 14% |
| I’m afraid that I might make my concussion symptoms worse by concentrating too much or being too mentally active.^b^ | 0% | 17% | 31% | 52% | 93% | 7% |
| I avoid thinking or concentrating too hard because it will elicit or increase the symptoms of my concussion.^b^ | 0% | 14% | 43% | 43% | 90% | 10% |
| I stop the activity I am doing when I sense my concussion symptoms are worsening. | 0% | 17% | 45% | 38% | 96% | 4% |
| Because of my symptoms most days I spend more time resting than doing cognitive activities. | 0% | 11% | 52% | 37% | 79% | 21% |
| **Social** | | | | | | |
| I avoid social settings that will make my symptoms worse. | 0% | 7% | 31% | 62% | 92% | 8% |
| I avoid catching up with friends and family because it will make my symptoms worse. | 0% | 7% | 52% | 41% | 100% | 0% |
| I avoid leaving my house because it will make my symptoms worse. | 0% | 11% | 34% | 55% | 100% | 0% |
| I avoid busy environments because it will make my symptoms worse.^c^ | 0% | 11% | 32% | 57% | 100% | 0% |
| I avoid going to the shops because they will make my symptoms worse.^c^ | 0% | 14% | 38% | 48% | 100% | 0% |
| I avoid interacting on social media because it will make my symptoms worse. | 0% | 18% | 41% | 41% | 100% | 0% |
| **Work** | | | | | | |
| I avoid physical jobs because it will increase my concussion symptoms. | 3% | 0% | 52% | 45% | 96% | 4% |
| I fear that my concussion symptoms will result in poor workplace performance. | 0% | 3% | 59% | 38% | 100% | 0% |
| I fear that my concussion symptoms will cause me to lose my job. | 0% | 6% | 45% | 49% | 100% | 0% |
| I should not do my normal work with my present symptoms. | 0% | 10% | 38% | 52% | 96% | 4% |

^a^ Removed as not all participants will drive a car so not applicable to all individuals who have sustained a concussion.

^b^ Removed as it is similar to “When I have my concussion symptoms, I’m afraid that thinking/concentrating too hard will make the symptoms worse.”

^c^ Removed as it is similar to “I avoid certain environments (bright, loud, fast-moving) because they worsen my symptoms.
